# Investigating Optimal Confinement Behaviour of Low-Strength Concrete through Quantitative and Analytical Approaches

**DOI:** 10.3390/ma14164675

**Published:** 2021-08-19

**Authors:** Mujahid Ali, Sheraz Abbas, Bashir Salah, Javed Akhter, Waqas Saleem, Sani Haruna, Shah Room, Isyaka Abdulkadir

**Affiliations:** 1 Civil and Environmental Engineering Department, Universiti Teknologi PETRONAS, Perak 31750, Malaysia; 2 Civil and Architecture Engineering Department, City University of Science and Information Technology, Peshawar 25000, Pakistan; sherazabbas@cusit.edu.pk; 3 Department of Industrial Engineering, College of Engineering, King Saud University, P.O. Box 800, Riyadh 11421, Saudi Arabia; 4 Department of Mechanical Engineering, University of Engineering and Technology Taxila, Rawalpindi 47080, Pakistan; javedakhter.uet@gmail.com; 5 Department of Mechanical and Manufacturing Engineering, Institute of Technology, F91 YW50 Sligo, Ireland; Saleem.waqas@itsligo.ie; 6 Civil Engineering Department, Bayero University Kano, Kano 700241, Nigeria; sharuna.civ@buk.edu.ng (S.H.); iabdulkadir.civ@buk.edu.ng (I.A.); 7 Department of Civil Engineering Technology, University of Technology, Nowshera 24100, Pakistan; shahroom@uotnowshera.edu.pk

**Keywords:** confinement behaviour, quantitative analysis, numerical modeling, low strength concrete, axial compression, stress–strain

## Abstract

Reinforced concrete is used worldwide in the construction industry. In past eras, extensive research has been conducted and has clearly shown the performance of stress–strain behaviour and ductility design for high-, standard-, and normal-strength concrete (NSC) in axial compression. Limited research has been conducted on the experimental and analytical investigation of low-strength concrete (LSC) confinement behaviour under axial compression and relative ductility. Meanwhile, analytical equations are not investigated experimentally for the confinement behaviour of LSC by transverse reinforcement. The current study experimentally investigates the concrete confinement behaviour under axial compression and relative ductility of NSC and LSC using volumetric transverse reinforcement (VTR), and comparison with several analytical models such as Mander, Kent, and Park, and Saatcioglu. In this study, a total of 44 reinforced-column specimens at a length of 18 in with a cross-section of 7 in × 7 in were used for uniaxial monotonic loading of NSC and LSC. Three columns of each set were confined with 2 in, 4 in, 6 in, and 8 in c/c lateral ties spacing. The experimental results show that the central concrete stresses are significantly affected by decreasing the spacing between the transverse steel. In the case of the LSC, the core stresses are double the central stress of NSC. However, increasing the VTR, the capacity and the ductility of NSC and LSC increases. Reducing the spacing between the ties from 8 in to 2 in center to center can affect the concrete column’s strength by 60% in LSC, but 25% in the NSC. The VTR and the spacing between the ties greatly affected the LSC compared to NSC. It was found that the relative ductility of the confined column samples was almost twice that of the unrestrained column samples. Regarding different models, the Manders model best represents the performance before the ultimate strength, whereas Kent and Park represents post-peak behaviour.

## 1. Introduction

Concrete is the most widely used construction material in all civil engineering projects across the world. A manufactured product, concrete is composed of cement, fine and coarse aggregate, water, and admixture(s) [1]. Concrete is relatively strong in compression and weak in tension; thus, the failure mode becomes brittle [2]. It is essential to know the properties of concrete and its use worldwide because most engineering and non-engineering projects are based on concrete structures. Instead of high and standard strength concrete (HSC) (>7000 psi), most of the engineering projects in Pakistan and other developing countries are from normal- and low-strength concrete (NSC (3000 to 5000 psi) & LSC (2900 psi)) [3]. According to the American Concrete Institute (ACI-318-08), a concrete structural member’s strength should be not less than 3000 psi [4].

A column is a compressive structural member that takes all the vertical load from the slabs and beams and safely transfers it to the ground. Columns are also designed to resist lateral forces that come from the wind and earthquakes. Additionally, columns are frequently used to support beams, slabs, or arches on which the upper part of the wall or ceilings rests [5]. A column is a critical structural member in a structure, and its failure may lead to the collapse of the whole structure [6,7]. The stability of the column plays a crucial role in structural stability. It has been documented that the axial capacity and relative ductility of an Standard Strength Concrete (SSC) and HSC used in reinforced cement concrete (RCC) columns of a structure are improved by the use of tightly braced lateral transverse steel anchors [8,9]. Different techniques were used to improve the stability, axial capacity, and ductility of reinforced columns. Steel bars are used with concrete to improve a structural member’s quality in tension and compression, which are the essential requirements of a structure [10,11].

The work on confinement concrete was started in the early 1900s. For the first time, the concrete strength improvement with the closing effect was initially reported by the Consideration in 1903 for concrete with NSC [12,13]. After investigating concrete confinement, most of the researchers work on the analytical equations without proposing stress–strain curves. The modelling effect of confinement was experimentally investigated for the first time by Richart et al. in 1928 [14,15], and the introduced analytical model by Richart et al. 1928 was modified by Balmer (1949) [16]. Instead of a circular section, the rectangular segment of the confinement reinforcement in the reinforced concrete was investigated by King (1946) and Balmer (1961) [17,18]. The stress–strain relationship was proposed by Soliman & Yu (1967) and Sargin (1971) for the confinement concrete [19,20]. Mander (1988) investigated circular, spiral, and ties lateral reinforcement for both cyclic and monotonic loading. The model was modified for both the static and dynamic loadings in early 1988 by Mander et al. [21]. The change in length of concrete concerning longitudinal compression and the energy balance stress–strain curve equation in the model was derived from the Popovics (1973) [22].

When a column is loaded with an external force parallel to its central axis, the external force is called the axial loading force. Axial loads are classified into concentric and eccentric axial loads [23]. This study is limited to concentric axial loading on concrete column samples. When an external axial compressive force acts directly along the structure’s central axis, the compressive force causes a single compression of the column without any bending moment, known as axial concentric load forces [24]. However, if the external axial compressive force is offset relative to the central axis, it will cause an eccentric axial load force [25]. When an external axial load was applied to the column, it is resisted by its internal molecular structure. The internal resistance to deformation of a structure is known as stress [26].

During a concentric axial compressive load, the centre concrete shortens in the longitudinal direction and expands in the lateral direction due to the effect/action of poison ratio [27]. Core concrete tends to bulge out, which produces pressure on the lateral reinforcement of the column. This lateral pressure on the tie will generate stress on the core concrete and keep the core concrete in a constrained state until the longitudinal steel bars yield or the transverse tie fracture [23]. The confinement in close spacing ties provided through transverse reinforcement and the coiled helix is considered a passive restraint [28,29,30]. This lateral restraint can be provided externally using fiber-reinforced polymer (FRP). Numerous features affect the confinement pressure, such as ties spacing/the distance of the connections, yield strength/flow resistance of the longitudinal bar, the longitudinal bar’s size, and the nature of transverse reinforcement [31,32,33,34,35].

HSCs have been used in multi-story buildings and long-span bridges to reduce structural element size and take up little space. However, the stress–strain curve of the HSC is a relatively steep and short post-peak branch which become more brittle than the NSC and LSC. Most HSC columns with a concrete cylindrical strength of 100 MPa could be very brittle if sufficient confinement reinforcement was not provided [36]. Most engineers mainly focused on the potential strength and paid little attention to ensure that the HSC have adequate ductility. HSC columns under axial load are generally more brittle than NSC columns. Therefore, HSC and NSC’s ductility and axial compression strength become a major concern [37]. The ductility of the HSC should not be less than the NSC/LSC structures.

The confinement effect of lateral transverse steel cannot be judged based on analytical models for low strength concrete. [38] suggested that most of the 2005 earthquake failure was due to low quality and concrete strength. In underdeveloped countries (Pakistan), local concrete construction methods are typical for lower strength than ACI-318-014 design standard, mainly due to less machine use, inadequate inspection, lack of skilled labor, and lack of knowledge and awareness [4,39]. The outcome of low concrete construction can be seen in the 2005 earthquake, which damaged structures such as 6300 educational institutes, 796 health units, and 600,000 houses in which a relatively average percentage was prefabricated reinforced concrete structures. Most of the columns were failed due to low ductility and cannot achieve maximum elongation. Meanwhile, in the 2005 earthquake, some designs were spoiled due to poor detailing of the beam’s column connection and poor concerting [40]. Based on the comprehensive review of Pakistan’s northern region, the concrete in the column was determined to have a strength of 2000 psi [3], and there was more distance between the anchors.

In previous decades, the utmost scientists and researchers have worked on various methods to advance concrete performance and ductility [41,42]. However, the researcher developed transverse steel confinement to enhance concrete performance and relative ductility [21,43]. The confinement behaviour is a complex phenomenon, and numerous researchers have investigated it experimentally and analytically. Previous research has clearly shown the confinement behaviour for the ductility improvement [44] and axial compression of standard and high-strength concrete columns [45,46,47,48] with and without the use of additives [49,50,51,52]. However, recent and past studies have rarely investigated the confinement behaviour of low-strength concrete using different ties spacing and grades of reinforcement. The current study uses different grades of volumetric transverse reinforcement (VTR) with different spacing to evaluate the NSC and LSC’s confinement behaviour under axial compression and relative ductility. Numerous features affect the confinement pressure, such as ties spacing/the distance of the connections, the yield strength/flow resistance of the longitudinal bar, the longitudinal bar’s size, and the nature of transverse reinforcement. Several analytical models were proposed to predict axial capacity and ductility of confined NSC and HSC columns [21,53,54,55]. However, there is a lack of research on the analytical models for low-strength concrete confinement behaviour. This research work is put forward to experimentally investigate low-strength concrete behaviour confined with close ties spacing to overcome this deficiency and select the best analytical model that predicts low strength concrete behaviour confined with compact transverse steel.

Previous research has experimentally investigated SSC and HSC’s confinement behaviour and validated it analytically with several analytical models [56]. However, recent and past studies have rarely evaluated NSC and LSC’s confinement behaviour under axial compression and relative ductility. The current research focuses on the experimental investigation of LSC confinement behaviour and validates it with several analytical models to choose the adequate model for predicting LSC under axial compression. Moreover, a total of forty-four (44) reinforced and unreinforced column specimens at a length of 18 in with a cross-section of 7 in × 7 in were prepared. Three columns of each set were confined with 2 in, 4 in, 6 in, and 8 in c/c lateral ties spacing. Universal Testing Machine (UTM) at a capacity of 200 t was used for the axial compressive strength and relative ductility.

## 2. Materials and Methods

**Figure 1:**
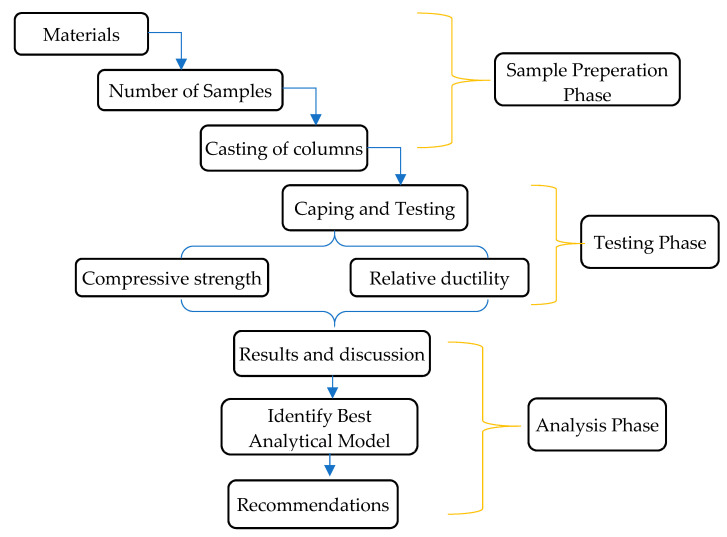
Methodology Flow Chart.

Most of the research work is performed on the analytical model of SSC and HSC performance, but minimal work is carried out on LSC (2000 psi) and NSC (3000 psi). With the absence of experimental work, it is impossible to use the same theoretical model as standard and HSC to determine low-strength concrete performance subjected to axial compression. An in-depth study/research is required to find LSC confined performance under compression restricted with the least spacing of transverse reinforcement. The current research mainly focuses on tie spacing, volumetric transverse ratio, relative ductility, and concrete strength. The flowchart shown in Figure 1 illustrates the research methodology Flow Chart. Materials that are used for the preparation of the mix design are discussed below.

### 2.1. Materials

Different materials were used in the mix design and concrete column specimens, such as fine and coarse aggregate, hydraulic cement, and reinforcement. As per ASTM C136-14, the results obtained from the sieve analysis of fine aggregate are presented in Table 1 [57]. The finesse modulus was 2.73, whereas the specific gravity was 2.65 as per ASTM C128-15 [58]. Meanwhile, the sieve analysis for coarse aggregates was carried out under ASTM C136-14 [57]. The bulk density of coarse aggregate was 102.68 lb/ft^3^, whereas the specific gravity is 2.69, and the absorption rate was 0.53 per unit by weight as per ASTM C128-15 [58]. However, the elongation and the flakiness were 11.26% and 8.3%. In contrast, several tests were performed on type-1 hydraulic cement to characterize the cement as per ASTM C150-20 [59]. As per ASTM C150-20, the finesse of cement was 88.5%. Using the Vicat needle test to determine the initial and final setting time of cement as per ASTM C191-19 [60], that was 90 and 229 min.

In this research, deformed bars were used as longitudinal and transverse reinforcement. Before using steel as a reinforcement, it is necessary to evaluate and know a material’s properties. UTM has a capacity of 200 t was used to find the reinforcement’s tensile and bending properties. In all column specimens, the deformed steel bars of 4 bars #4 bar with a grade of 60 were used as longitudinal reinforcement. Two different randomly deformed steel bars of #3 and #2 bars were used for the transverse reinforcement in the concrete columns. For the tensile strength of the randomly selected transverse and longitudinal reinforcement, the UTM capacity of 200 t was used. The results obtained from the UTM are represented in Table 2 and Table 3.

### 2.2. Mix Design of Normal Strength Concrete and Low Strength Concrete

Mixture proportioning is a process of selecting suitable ingredients and determining their relative proportions with the objective of producing concrete with a certain minimum workability, strength, and durability as economically as possible. The theoretical experimental method was used to determine the recipe of the concrete mix. The design of the concrete mixture began with determining the qualitative features of components and determining their basic properties. On the basis of general formulas, the proportions of aggregates were selected, followed by the amount of individual concrete components. Using the properties of fine and coarse aggregate and cement as mentioned above, the mix design of concrete was carried out for 28 days of 3000 psi strength. The concrete mix ratio was taken by its weight of 1:2.28:2.76 (cement: fine aggregate: coarse aggregate), with a w/c of 0.57. The volume of one cylinder is 0.0144 m^3^, whereas the mix proportion of cement is 238.41 kg/m^3^ (3.43 kg), fine aggregate is 603.96 kg/m^3^ (8.64 kg), and coarse aggregate is 822 kg/m^3^ (11.84 kg) for a single column. Using a 0.57 w/c ration, the proportion of water is 1.955 L. Twenty-two NSC and LSC casing specimens were cast in two sets of three, each with the same size, having the same longitudinal but different transverse reinforcement. The slump ranged from 1–2 in, and the absorption rate of coarse aggregate was 0.53 per unit by weight. In contrast, the fine aggregate’s specific gravity and fineness modulus were 2.73 and 2.69, respectively. As per ASTM C39/C39M-20 [61], three cylinders were cast and tested for 7 and 28 days. Similarly, the mix design for the LSC of 2000 psi was taken by its weight of 1:2.92:3.28 (cement: fine aggregate: coarse aggregate) and with a w/c of 0.66.

### 2.3. Preperation, Casting and Curing of Specimens

A column specimen with a length of 18 in and a cross-section of 7 in × 7 in was designated for the concentric axial compression. Each sample is reinforced with longitudinal/vertical bars of 4 bars #4 bars. The horizontal/transverse reinforcement is provided in square hoops with variable ties spacing #3 and #2 bars. Due to the limited height available between the two jaws of the Universal Testing Machine (UTM) loading unit, the height of the column specimen was kept at 18 in. Column specimens were fabricated and cast for mix designs of 3000 psi (NSC) and 2000 psi (LSC) cylinder compressive strength. The column specimen details and terminology are given in Table 4 and Table 5.

**Figure 2:**
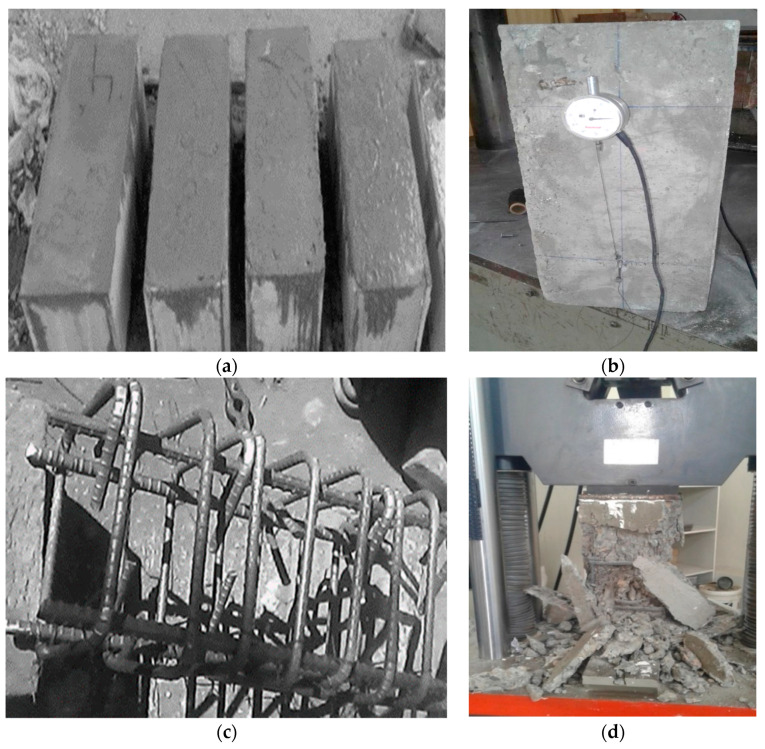
(**a**) Casting, (**b**) instrumental setup, (**c**) steel cages (**d**) crushed samples after the compressive strength test.

Mixed designs were used for concrete strength of 2000 and 3000 psi. The steel cages were placed in the formwork with a transparent cover of 3/4 in between the centre line of rectangular hoops at the formworks’ inner surface. The columns were cast in three layers, and each layer was vibrated before placing the next layer through a vibrator. The casting of the concrete column specimens is shown in Figure 2a.

After casting the sample, all concrete columns and cylinders are covered with a wet hessian sack for thoroughly moist curing. After the curing, the concrete columns were instrumentally set up and placed in the UTM for testing.

### 2.4. Instrumentation and Testing of Column Specimen

The testing setup arrangement is a critical stage to acquire accurate data from testing column specimens. Each column sample was fully equipped and arranged to collect the desired data. For longitudinal deformation of the column sample, two displacement transducers with a capacity of 60 mm were connected on the front and rear face sides of the column sample to achieve the deformation on both sides. The calibration length was 9 in on both sides of the sample. Displacement sensors were fixed in each face’s middle, as shown in Figure 2b. The record date comprises the axially applied load and longitudinal deformation due to the applied load on the column specimen. Further visible observations were recorded with the camcorder while testing the column samples.

As shown in Figure 2c, the confined column specimens were tested in the Engineering material lab at the University of Engineering Technology (UET), Peshawar, Pakistan. A UTM with a capacity of 200 t was used for the application of monotonic axial compressive load. For the recording of load and displacements in real time, the National Instruments LabVIEW data acquisition system was used.

Both sides of the column specimen were caped with plaster of Paris with a depth of 12 mm to distribute the load on the column specimen uniformly. Furthermore, for the uniform distribution of loads, a steel plate of cross-sectional 8 in × 8 in was placed on the column specimen’s upper surface. The deformation gauges with a 60 mm capacity were installed on both sides to record the deformation before spalling the concrete cover. The gauge length for each displacement was kept at 9 in and was calibrated and connected with the data acquisition system.

The samples were strictly aligned with the UTM loading unit to certify concentric axial loading on the sample. The loading rate was 20–50 psi and remained constant throughout the test. The load continues until the column sample collapses due to the longitudinal bars’ compliance or rupture of the transverse hoops. After testing the column samples, the data were documented and saved in a system for further analysis.

As the load advanced to the peak/ultimate value, the cracks appeared on all sides of the column specimen, as shown in Figure 2d. It was detected that the confined column specimen’s behaviour was quite peculiar compared to the unconfined column specimen’s behaviour. Meanwhile, the load dropped to a certain value when spalling concrete occurs and increased again, especially in the closed concrete column pattern. From the test results, it was observed that unconfined column samples failed due to yielding/deformation of longitudinal bars, while the confined column samples failed due to rupture/breakage of transverse hoops/rings. To calculate and report the CS (Compressive Strength) of each specimen, refer to Equation (1).

(1) $$\mathrm{Compressive}\;\mathrm{strength}\;\left(\mathrm{CS}\right)=\frac{W}{A}$$

where the CS measured in psi, *W* = load in a pound (lb), *A* = average of the gross area of the upper and lower bearing surfaces of the specimens (in^2^).

Table 6 shows the cylinder strength for the 3000 psi by using a concrete mix ratio was 1:2.28:2.76 by weight and 0.57 w/c ratio. The cylinder’s average strength for 7 and 28 days was found to be 2271 psi and 2978 psi, respectively. The recent studies found that the maximum (99.9%) CS can be achieved at 28 days of curing for the NSC and LSC; therefore, the scope of the study was limited to 28 days [39]. Meanwhile, the cylinder strength for the 2000 psi by using a concrete mix ratio was 1:2.92:3.28 by weight, and 0.66 w/c ratios are shown in Table 7. Average concrete strength for 7 days and 28 days was found at 1504 psi and 1976 psi, respectively.

A column’s ductility is greatly influenced by the concrete strength, the ratio of steel in longitudinal and transverse directions, axial load, and the distance between the ties. All of these factors must be considered before evaluating the ductility of columns. When the axial load level is higher than the balanced axial load level, the column becomes a compression failure.

### 2.5. Analytical Models

The researcher has proposed several analytical equations based on the experimental results to forecast concrete confinement behaviour. The work on the confined concrete was started in the early 1900 century. Considere initially reported the strength enhancement in concrete with the confinement effect in 1903 for the normal strength of concrete. Later on, Richart et al., in 1929 [14], experimentally investigated the strength of concrete columns due to lateral confinement. After these studies, most of the work in this direction has been completed. No stress–strain curve has been proposed; only analytical equations were presented. This model was in a series of short column specimen tests.

**Figure 3:**
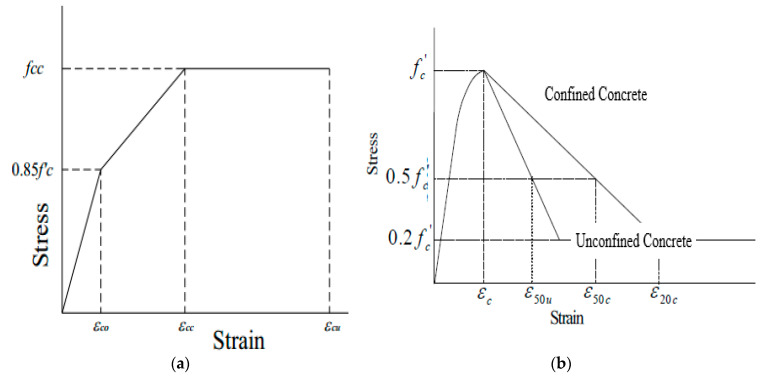
(**a**) Proposed model by Blume et al., 1961 (**b**) Proposed curve by Kent and Park.

Blume et al., 1961 [18], introduced the influence of yield strength in various analytical equations for describing the confinement behaviour of normal concrete. In their analytical model, the stress–strain curve consisted of three parts; *ε*_*co*_, *ε*_*cc*_, *ε*_*cu*_ show the strain, whereas *f_cc_* and 0.85*f′_c_* show the stress as shown in Figure 3a. The analytical equation is given by Equations (2)–(5).

(2) $$f_{cc}=0.85{f^{\prime }}_{c}+4.1\frac{A_{\mathrm{cc}}}{sh}$$

(3) $$\varepsilon_{co}=\frac{0.22{f^{\prime }}_{c}+400\;\mathrm{psi}}{10^{6}\;\mathrm{psi}}$$

(4) εco=5 εy

(5) εcu=5 εsu

where *f_cc_* is the maximum concrete stress and *ε*_*cc*_/*ε*_*cu*_ is the corresponding strain in the Blume et al. model [18]. However, *f_c_* is the longitudinal compressive concrete stress, and *f′_c_* is the compressive strength of confined concrete, whereas *ε_c_* is the longitudinal compressive concrete strain.

In 1967, Soliman and Yu [19] developed a model that focused on the geometry of the cross-section, ties spacing, effectiveness of ties, and the area of steel used for the ties. However, in 1971, Kent and Park [62] introduced a model in which they assumed that the maximum capacity was the same for confined and unconfined concrete. The stress–strain curve starts from the origin to ultimate compressive strength. The confined concrete curve is of a straight line descending from the peak strength followed by a flat line showing unlimited deformation capacity. In the unconfined concrete curve, the descending part curve is in a straight line, but a smaller amount straining capacity the proposed curve is shown in Figure 3b.

Equation (6) is for the ascending:

(6) fc=f′c[2 εc εco−( εc εco)2]

Equation (7) is for the descending:

(7) $$f_{c}={f^{\prime }}_{c}\;\left[1-z\left(\varepsilon_{c}-\varepsilon_{\mathrm{co}}\right)\right]$$

Manders et al., 1988 [21], proposed a model based on the same concept put forwarded by Shiekh and Uzumeri (1982) [55]. As the confining lateral pressure was based on the cross section’s geometry, this model was suitable for any cross-section [54]. One of the advantages of the Manders model is the combination of both static and dynamic load types. Manders adopted the energy balance method in the stress–strain curve model to measure the strain in the concrete in longitudinal compression and were based on the research of Popovics 1973 [22] in which he derived a stress–strain equation for the longitudinal compression in concrete.

## 3. Results and Discussion

### 3.1. Compressive Strength of LSC and NSC

**Figure 4:**
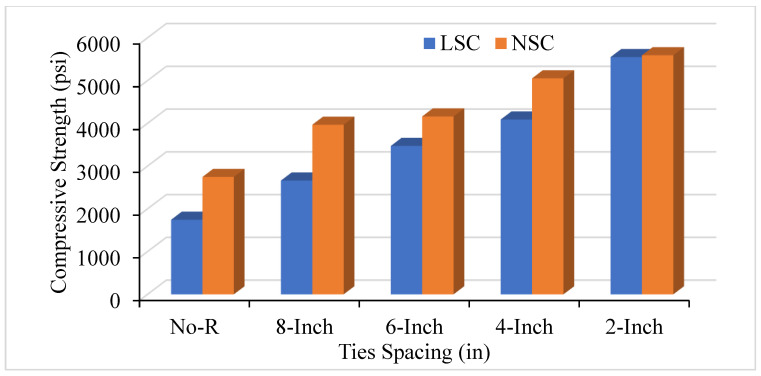
Compressive strength of Low Strength Concrete (LSC) and Normal Strength Concrete (NSC) with different ties spacing of #3 bar.

Experimental results illustrate that decreasing the amount of spacing between the ties will increase the axial CS in both the NSC and LSC. Figure 4 shows that when there is no reinforcement in the concrete column, the strength ranges from 1500 to 2500 psi which is the only strength of concrete and longitudinal bars; however, the strength increases by providing the transverse bars and decreasing the spacing between them. Reducing the ties’ spacing increases the number of ties in the concrete columns and leads to increased CS and relative ductility in both the NSC and LSC. The 8 in c/c spacing shows the CS of 2662 psi in LSC, and 3967 psi in NSC, whereas the 2 in c/c offer the highest CS of 5550 psi in both NSC and LSC. As the LSC should not be less than 2500 psi and the HSC is above 5000 psi, the current study’s benchmark was selected as 2500 psi to 5000 psi. Moreover, the present study follows the HSC and SSC and compares the results with several analytical models, such as Mander and Kent models. Therefore, the analytical models were the benchmark for the current study.

**Figure 5:**
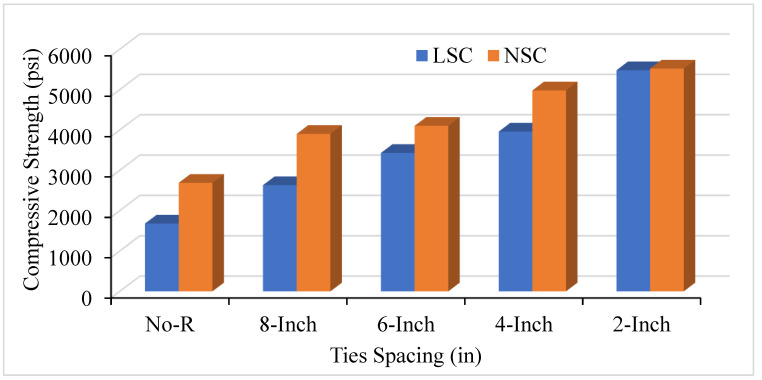
Compressive strength of LSC and NSC with different ties spacing of #2 bar.

**Figure 6:**
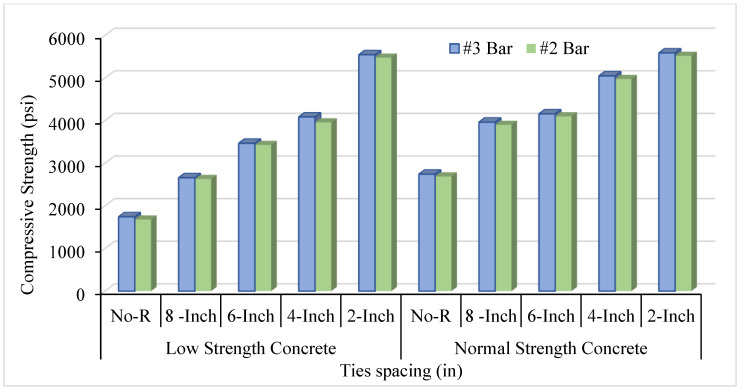
Comparison of axial strength of LSC and NSC with different Volumetric Transvers Reinforcement (VTR).

Furthermore, volumetric transverse reinforcement (VTR) also affects axial compression and relative ductility. Changing the bar number from #2 to #3 positively affects and increases the concrete columns’ CS and ductility. Figure 5 compares LSC and NSC’s CS with a ties spacing of #2 bar. When comparing the LSC core strength with the NSC core strength, it was observed that the lateral steel confinement effect was more effective with the LSC compared to the NSC. The results in unconfined column specimens were practically the same, whereas LSC’s core strength was double the NSC central strength of the confined column. The bar number used for the confinement and the ties’ spacing between the transverse reinforcement greatly affects the CS. Figure 6 illustrates the comparison of VTR of #2 and #3 and the spacing of the ties between transverse reinforcement. As the number of spacing decreases between the ties and increases the bar number, improve the concrete cylinder’s CS in both NSC and LSC. However, NSC shows better CS than LSC in terms of ties spacing and VTR because of the difference in the mix design ratio of the NSC and LSC.

### 3.2. Realtive Ductility of LSC and NSC

**Figure 7:**
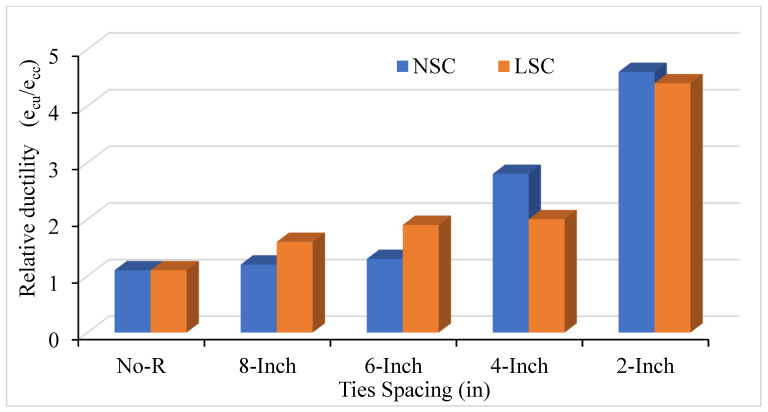
Relative ductility of LSC and NSC with different ties spacing of #3 bar.

**Figure 8:**
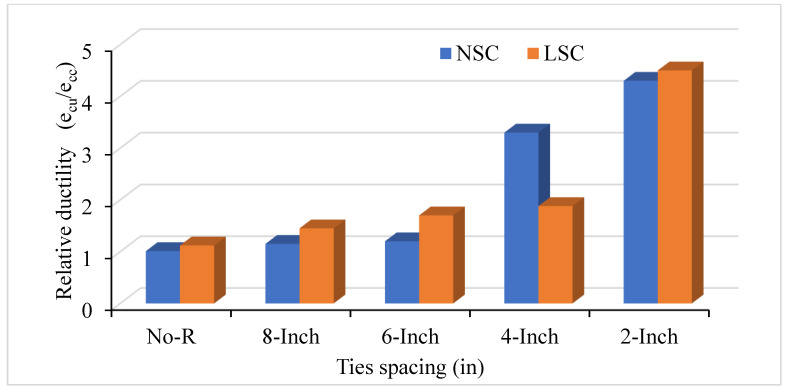
Relative ductility of LSC and NSC with different ties spacing of #2 bar.

**Figure 9:**
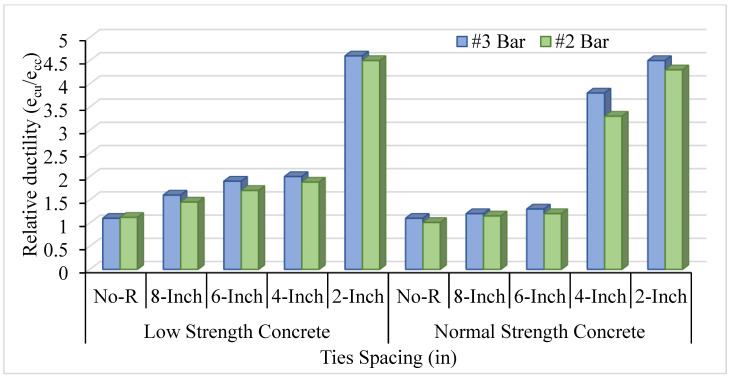
Comparison of relative ductility of LSC and NSC with different VTR.

The ratio of crushing strain to the peak strain is defined as relative ductility. The bar number and spacing between ties affect concrete columns’ relative ductility. Figure 7 shows LSC and NSC’s relative ductility with different ties spacing of #3 bar, whereas Figure 8 shows this for the #2 bar. Moreover, Figure 9 compares the relative ductility of LSC and NSC with different VTR and ties spacing. The VTR of #3 bar with a spacing of 2 in c/c shows the greatly enhanced concrete columns in both LSC and NSC, whereas the low relative ductility was found in the LSC #2 bar with no transverse reinforcement. Similarly, with core compressive strength, the relative ductility is also greatly affected by the bar’s spacing than the size of the bar.

As the number of bars increases and the spacing between them decreases, it increases the relative ductility in concrete columns. The relative ductility at VTR #3 and #2 bar of LSC is more than the NSC in 8 in and 6 in c/c, but the opposite in 4 in c/c due to the effect of ties spacing and VTR. The spacing between the ties and VTR greatly affect the LSC relative ductility than that of the NSC. However, reducing the spacing between the ties at a maximum (4 and 2 in c/c) greatly affects the relative ductility of NSC, as shown in Figure 7. Compared to the NSC, the LSC shows high relative ductility, except for at 4 in c/c.

In comparing NSC and LSC at different VTR, the NSC shows better ductility in both #2 and #3 bars than LSC. The highest relative ductility was found in the LSC with 2 in c/c of VTR #3 bar, which means that the VTR and the bar spacing greatly affected the LSC. However, overall, the NSC’s relative ductility at both #2 and #3 bar shows high ductility, as shown in Figure 9. As a 2-inch increase in the ties spacing increase two times the relative ductility in NSC and LSC.

### 3.3. Stress–Strain Curve of NSC and LSC for Various Confinement Spacing

**Figure 10:**
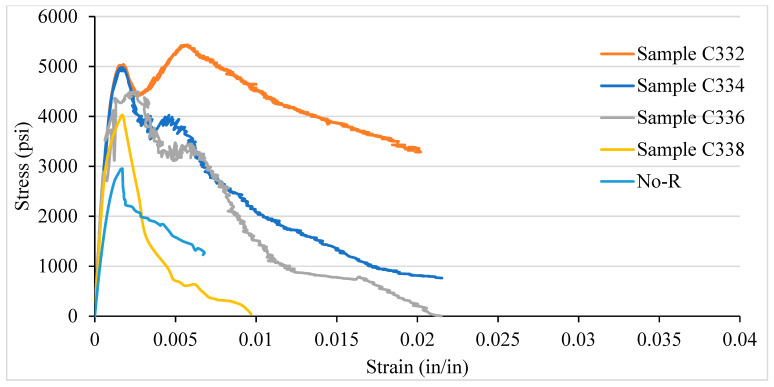
Stress–strain curve of NSC for various confinement spacing.

**Figure 11:**
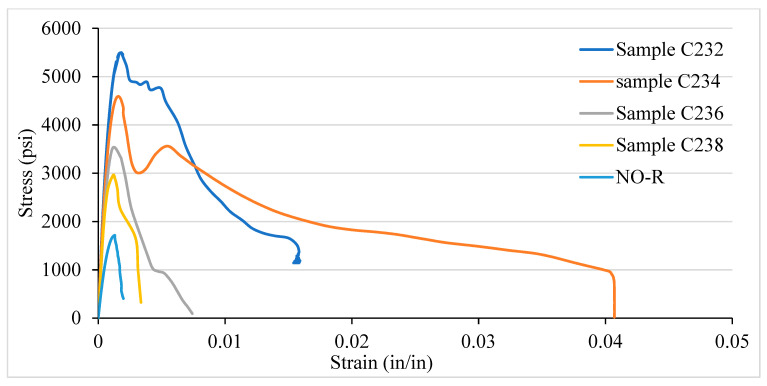
Stress–strain curve of LSC for various confinement spacing.

In order to study the effect of confinement and ties spacing on confined concrete behaviour, we compare the response curves of the confined concrete, as shown in Figure 10 and Figure 11. The stress–strain curves were drawn after the data were obtained from the data acquisition system. The stress–strain curve provides multiple information on different points, which are: elastic limit, yield point, strain hardening, ultimate strength, necking, peak point, and fracture. Figure 10 illustrates the stress–strain curve for the VTR of #3 bar with a spacing of 2, 4, 6, and 8 in c/c and no reinforcement for NSC. The concrete column with a spacing of 2 in c/c shows the highest CS of 5038 psi with a strain of 0.0017 at elastic limit whereas, the ultimate strength is 5432 psi at a strain of 0.005 which give a modulus of elasticity (stress/strain) 1,086,400 psi (7.49 GPa). In addition, the fracture strength of 2 in c/c was near to the peak/ultimate strength of the 8 in c/c and was more significant than the columns with no reinforcements, as shown in Figure 10. As the amount of spacing between the ties increases from 2 in c/c to 8 in c/c, the CS decreases. Further, the column with no reinforcement shows the lowest CS and becomes fractured with minimal strain changes.

The core concrete stresses are greatly affected by decreasing transverse steel spacing compared with the bar’s size. The core stresses in LSC are twice the core stresses of NSC. In contrast, the ductility of NSC and LSC remains approximately the same.

Figure 11 illustrates LSC’s stress–strain curve for the VTR of #3 bars and ties spacing of 2, 4, 6, and 8 in c/c and no reinforcement. Like NSC, the LSC shows the highest strength at 2 in c/c. However, LSC’s strength is relatively higher than NSC’s strength, which is 5497 psi at a strain of 0.0017. The modulus of elasticity in LSC is 3,233,529.4 psi, equal to 22.29 GPa, and is higher than the modulus of elasticity of NSC, but the fracture point occurs at a strain of 0.015. Besides the columns having 4 in c/c, ties spacing shows the highest strain at a fracture point. Compared to NSC, the LSC shows the highest strength only at 2 in c/c, whereas the rest are the opposite. After the ultimate strength, the NSC takes sufficient loads and sustains for a great strain until the fracture, whereas the LSC reaches the ultimate with a very little strain and goes to fracture, which behave like a brittle material. Additionally, both the NSC and LSC come under the ASTM and ACI standards.

### 3.4. NSC Vs Analytical Stress–Strain Curve for Various Confinement

After testing the concrete columns, the experimental results were compared with the analytical models introduced by Mander et al. in 1988 and Kent and Park in 1972. As different types of confinement were used with different ties spacing, the graphs are consequently different. A comparison was made among the specimens with 8-, 6-, 4-, and 2-in tie spacing column specimens. It has been observed that the relationship suggested by various analytical models are to address the ultimate strength and strain increment of the concrete stress–strain curve. The comparisons conducted here with numerous experimental data provide an adequate correlation with a wide range of relevant parameters. It turns out that a well-generalized relationship works, especially for confined-core concrete with low concrete strength.

**Figure 12:**
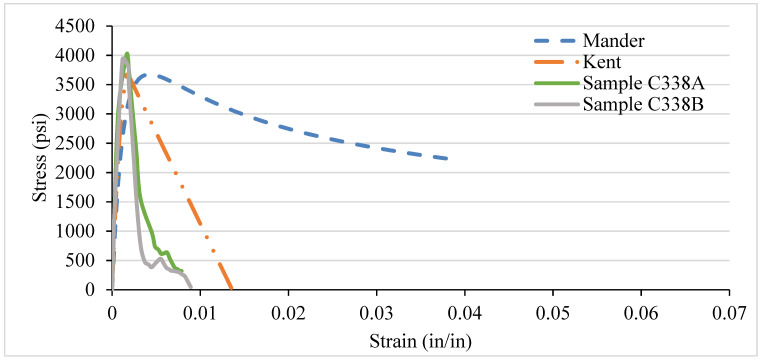
C338 NSC Vs Analytical Model (8 in c/c).

**Figure 13:**
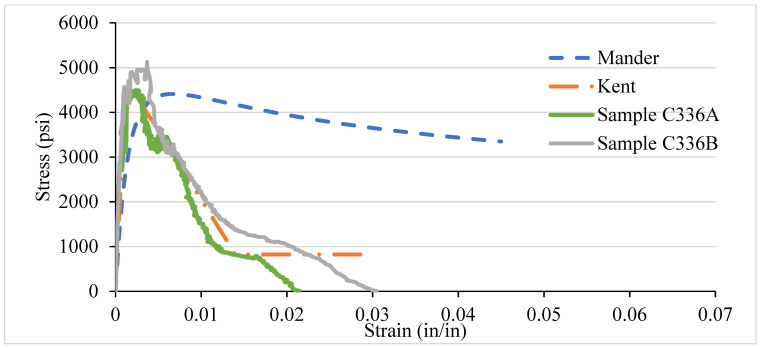
C336 NSC Vs Analytical Model (6 in c/c).

**Figure 14:**
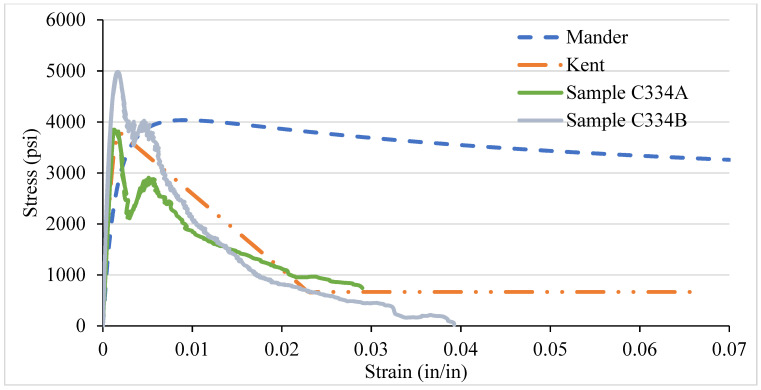
C334 NSC Vs Analytical Model (4 in c/c).

**Figure 15:**
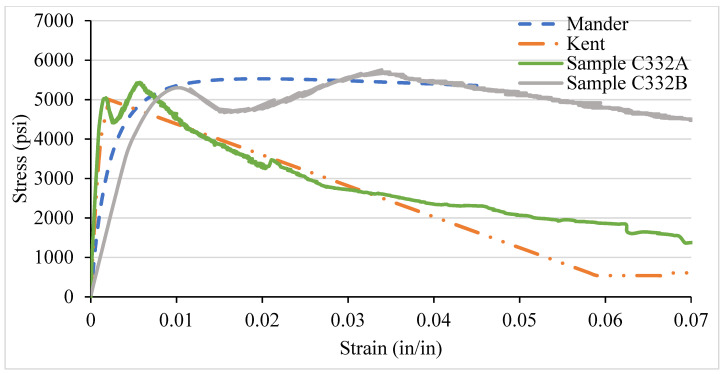
C332 NSC Vs Analytical Model (2 in c/c).

Figure 12, Figure 13, Figure 14 and Figure 15 show the comparison of NSC columns with analytical models using 8-, 6-, 4-, and 2- in c/c ties spacings. The Manders model best represents the performance before the peak strength, whereas the Kent and Park model best represents NSC’s performance after the peak strength. The concrete with 2-inch c/c ties spacings shows a smaller lateral expansion under the axial compression load than the 8 in c/c due to its higher modulus of elasticity. At the same strain for all the ties spacings (0.0016), the 8 in c/c shows the highest 4007 psi strength, whereas the 6, 4, and 2 in c/c show 4879 psi, 4914 psi, 4949 psi, respectively. As the spacing between the ties decreases from 8 in to 2 in c/c and increases the number of ties, the strength increases without changing the strain. However, a 0.003-in increase in the strain can affect concrete columns’ strength by 80% with a spacing of 8 in c/c, whereas 6, 4, and 2 in c/c affect it by 27.2%, 26.5%, and 9.76%. Initially, the concrete achieved high strength and reached peak/ultimate strength with a very small strain of 0.001 in/in, but after the peak, a small change in load produced a greater change in the original length. The Manders model best represents the elastic stage and sustains for a large change in length, whereas the Kent and Park model goes to fracture after the ultimate load.

Compared to 8 and 6 in c/c, the 4- and 2-inch c/c spacings show a large change in the original length and sustain for a long time without fracture. The lateral expansion in the 2 in is meagre due to the internal microcracking and the ties’ spacing. Test results show that significant strength and ductility can be achieved if lateral reinforcement is provided.

### 3.5. LSC Vs Analytical Stress–Strain Curve for Various Confinement

**Figure 16:**
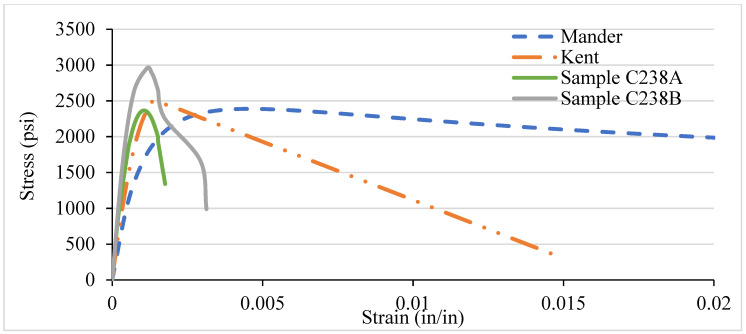
C238 LSC Vs Analytical Model (8 in c/c).

**Figure 17:**
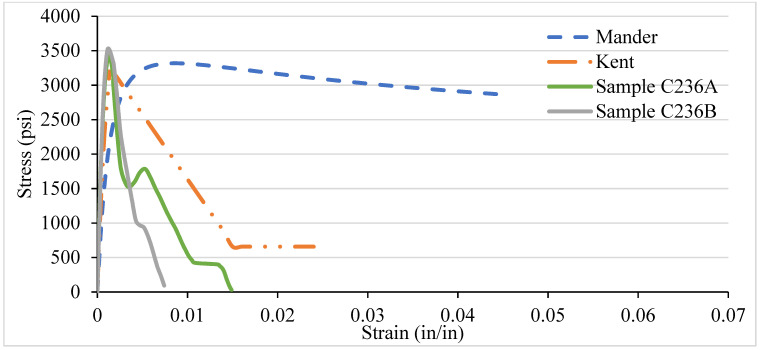
C236 LSC Vs Analytical Model (6 in c/c).

**Figure 18:**
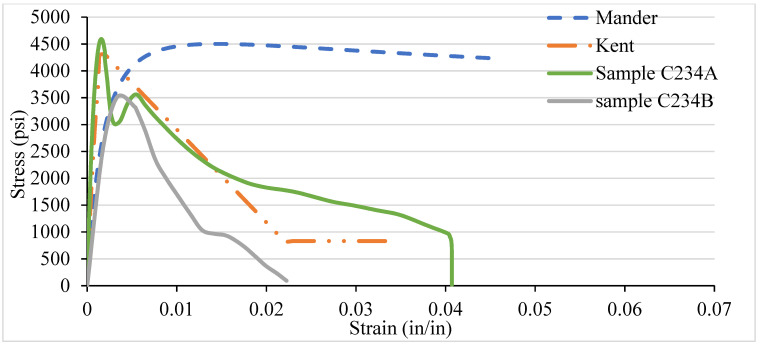
C234 LSC Vs Analytical Model (4 in c/c).

**Figure 19:**
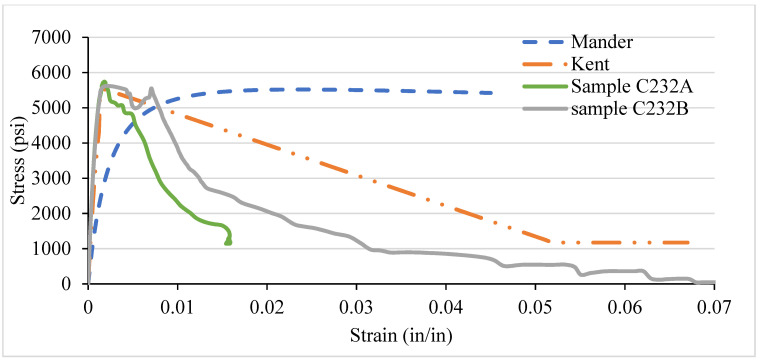
C232 LSC Vs Analytical Model (2 in c/c).

Likewise, the NSC, the different confinement, and the VTR of LSC were also compared with the analytical models. In LSC, the Kent and Park model best represents the core concrete strength’s performance before, during, and after the peak/ultimate strength until the fracture, as shown in Figure 16, Figure 17, Figure 18 and Figure 19. There are several reasons why there is a discrepancy between the analytical models and experimental results. The main reason is the current study adopted by LSC. In contrast, the analytical models are adopted for the NSC and HSC; that is why there is a variation in the curve lines of the experimental results and analytical models, especially the Manders model. Moreover, there is also a human and instrumental error in the experimental results, which shows the discrepancy. In addition, most of the concrete work done in Pakistan is for low-strength concrete. Further, those specimens that have more than 3000 psi strength follow the analytical models. Moreover, the strength of 8- and 6-inch c/c ties spacing in the LSC is relatively low compared with the NSC, whereas the strength in 4 and 2 in c/c is relatively higher than the NSC. Like NSC, the LSC also shows the highest strength in the 2 in c/c and decreases by increasing the spacing between the ties. The strength becomes more than double by increasing the spacing from 8 in c/c (2259 psi) to 2 in c/c (5600 psi) at the same strain.

Less lateral expansion due to the close confinement in 2 in c/c provides a high strength and modulus of elasticity. The spacing between the ties increasing will provide low strength and cause buckling in the column. The columns with closed ties of 2 in c/c go to the plastic limit and give warring before the fracture. In contrast, the columns with 6 and 8 in c/c suddenly fracture after achieving the peak/ultimate load, which is more dangerous in civil engineering projects. The lateral restraint pressure on the core concrete is directly related to the concrete columns’ lateral reinforcement. Thus, the larger confining pressure results in better confining efficiency. Figure 17, Figure 18 and Figure 19 shows the confinement of concrete columns using different spacing. Increases in strength from 2259 psi to 5600 psi are because of reducing the spacing of ties from 8 in c/c to 2 in c/c, which can affect the concrete column’s strength by 60%.

Compared with NSC, the LSC shows higher strength in 2 in c/c, whereas the rest are the opposite. Moreover, the NSC’s Manders model shows better performance before the ultimate strength, whereas the Kent and Park model offers the best performance after the peak strength. However, the Kent and Park model shows the best performance before and after LSC’s ultimate strength. Overall, it can be seen that there is a slight variation in the experimental and analytical model. In addition, both the models are validated experimentally.

## 4. Conclusions and Discussion

The current research experimentally evaluates the compressive strength and relative ductility of confined and unconfined NSC and LSC with varying VTR and ties spacing. In contrast, the experimental results were compared with the analytical models and select the best analytical model that best described low-strength concrete’s detention behaviour with low-pitch cross-sections. Based on the experimental results, the following conclusions can be drawn.

Experimental results show that the concrete core stresses are greatly influenced by reducing the transverse steel spacing. As the spacing between the ties increases, the strength of the concrete-reinforced column become decreases. The enhancement in the concrete core’s compressive capacity due to confinement was almost double in LSC confinement than in the NSC column specimens. Keeping VTR the same and by reducing the transverse steel spacing, the axial core compressive strength increases. Therefore, the confinement effect due to transverse steel is more in LSC compared to NSC.

Additionally, the axial compressive strength and relative ductility were greatly affected by the spacing between the ties in the reinforced concrete column. Moreover, the reinforcement grade used for the ties also affects the concrete compressive strength and relative ductility. Change in the bar number from #2 to #3 bar increases the compressive strength. Therefore, bar #3 shows the highest compressive strength than the #2 bar.

In terms of relative ductility, there was a slight variation in the concrete core’s relative ductility in NSC and LSC. The relative ductility was found to be almost equal for NSC and LSC by comparing the same VTR column specimens. It was found that the relative ductility of the confined column samples was almost twice that of the unrestrained column samples.

In comparing experimental results with the analytical models, for NSC columns, the behaviour of the concrete column before the peak is better described by the Manders model, whereas the Kent and Park model better describes the post-peak behaviour of the concrete column. However, there is a variation in behaviour of LSC columns. The Manders model shows high strength, whereas the Kent and Park model shows better behaviour before and after the peak. Overall, it can be seen that there is a slight variation in the experimental results and analytical models. In addition, both the Mander and Kent models are validated experimentally.

## Figures and Tables

**Table 1:** Gradation of fine aggregate.

Sieve No. (ASTM)	Sieve Opening (mm)	Passing Weight (g)	Total Weight (%)	Retain (%)	Passing (%)
No. 4	4.75	0	0	100	0
No. 8	2.36	4	0.689	99.31	0.69
No. 16	1.18	110	18.96	80.35	19.65
No. 30	0.60	218	37.58	42.77	57.23
No. 50	0.30	203	35.00	7.77	92.23
No. 100	0.15	45	7.75	0.02	99.98
Total weight	-	580	-	-	2.73

**Table 2:** Tensile properties of longitudinal reinforcement.

S. No.	Bar Diameter	Yield Strength (psi)	Ultimate Strength (psi)	Percentage Elongation	Effective Dia. (in)	Weight (lb/ft)	Yield Strength (Mean)	Standard Deviation
1	#4	71,036.5	89,105.07	16.40	0.48	0.595	70,705.45	265.90
2		70,178.3	88,336.32	17.19	0.48	0.600		
3		70,901.5	88,873.09	16.40	0.48	0.598		

Dia stand for Diameter, whereas the # shows the bar number.

**Table 3:** Tensile properties of Transverse reinforcement.

S. No.	Bar Diameter	Yield Strength (psi)	Ultimate Strength (psi)	Percentage Elongation	Effective Dia. (in)	Weight (lb/ft)	Yield Strength (Mean)	Standard Deviation
1	#3	70,036.5	88,305.07	15.90	0.48	0.595	70,898.76	266.60
2		72,178.3	86,936.32	16.89	0.48	0.600		
3		70,841.5	88,553.09	15.70	0.48	0.598		

Dia stand for Diameter, whereas the # shows the bar number.

**Table 4:** Description of the sample preparation.

Cylindrical Strength (psi)	Transverse Steel Bar #	Ties Spacing (in c/c)	No. of Specimens	VTR	Remarks
2000/3000	#3	2	6	0.021	confined
		4	6	0.0062	un-confined
		6	6	0.011	
		8	6	0.0047	
	#2	3	6	0.007	confined
		4	6	0.005	un-confined
	Un-confined	-	8	-	un-reinforced
Total	44 specimens				

**Table 5:** Nomenclature of column specimen.

Strength (psi)	Ties Bar	Spacing (in)	Codes	Code Details
2000	#3	2	C232	C = Column 2 = Concrete strength (2 ksi) 3 = #3 Ties Bar 2 = Spacing
		4	C234	
		6	C236	
		8	C238	
	#2	3	C233	
		4	C234	
3000	#3	2	C232	
		4	C234	
		6	C236	
		8	C238	
	#2	3	C233	
		4	C234	

**Table 6:** 7 days concrete compressive strength.

S. No.	Desired Strength (psi)	UTM Load (t)	Strength (psi)	Average Strength (psi)	Standard Deviation
1	2000	20.6	1606.302	1594.605	4.21
2		20.3	1582.909		
5	3000	29.8	2323.5	2378.262	8.13
6		31.2	2432.84		

**Table 7:** 28 days concrete compressive strength.

S. No.	Desired Strength (psi)	UTM Load (t)	Strength (psi)	Average Strength (psi)	Standard Deviation
1	2000	25.1	1842.77	1805.74	55.63
2		26.1	1800.93		
3		24.5	1754.16		
4		26.41	1825.10		
5	3000	38.14	2973.49	2978.94	84.08
6		38.8	3024.95		
7		37.9	2954.78		
8		38	2962.58		

## Data Availability

The data presented in this study are available on request from the corresponding author.
